# First time molecular detection and phylogenetic relationships of torque teno sus virus 1 and 2 in domestic pigs in Uganda: further evidence for a global distribution

**DOI:** 10.1186/1743-422X-9-39

**Published:** 2012-02-15

**Authors:** Matilda Brink, Karl Ståhl, Charles Masembe, Ademun Rose Okurut, Mikael Berg, Anne-Lie Blomström

**Affiliations:** 1 Section of Virology, Department of Biomedical Sciences and Veterinary Public Health, Swedish University of Agricultural Sciences, Uppsala, Sweden; 2 College of Natural Sciences, Makerere University, Kampala, Uganda; 3 Ministry of Agricultural Animal Industry and Fisheries, Entebbe, Uganda

**Keywords:** Torque teno sus virus (TTSuV), phylogenetic analysis, Uganda

## Abstract

**Background:**

Torque teno sus virus 1 (TTSuV1) and 2 (TTSuV2) are small, single-stranded circular DNA viruses belonging to the *Anelloviridae *family. Available studies clearly show that both viruses are widely distributed in the pig populations in America, Europe and Asia, although the impact of the infection is still unclear. Currently, the situation in domestic pig populations on the African continent is not known. Therefore, the aim of this study was to investigate the possible presence of the two viruses in domestic pigs in Uganda, and describe the phylogenetic relationships to those in the rest of the world.

**Results:**

Ninety-five serum samples from six districts in Uganda were used, and PCR using TTSuV1 and 2 specific primers for the UTR region was run for viral nucleic acid detection. The positive samples were sequenced, and phylogenetic analyses performed in order to compare the Ugandan sequences with sequences from other parts of the world. The prevalence of TTSuV1 and 2 in the selected domestic pigs were estimated at 16.8% and 48.4% respectively, with co-infection found in 13.7%. The sequence identity was 90-100% between the Ugandan TTSuV1; and 63-100% between the Ugandan TTSuV2 sequences.

**Conclusion:**

This is the first report on the presence of TTSuV1 and 2 in domestic pigs in Uganda. These results highlight the importance of screening for emerging viruses given the globalisation of human activities.

## Background

Torque teno virus (TTV) is a small non-enveloped, circular, single-stranded DNA virus belonging to the *Anelloviridae *family. It was first discovered in 1997 in a patient with post-transfusion hepatitis [1,2], but has since then been found in a number of species including chimpanzees, dogs, cattle and pigs [3]. The involvement of TTV in different diseases has been investigated but so far no clear connection has been shown; the role of TTV, if any, is complicated by the fact that the virus seems to be part of the normal viral flora in most investigated species [3-5].

TTV was first discovered in pigs in 1999 [6] but a retrospective study from Spain showed that the virus had been present at least since 1985 [7]. Genetic analyses distinguished two genogroups, TTV-1 and TTV-2 [8], which recently were proposed to be redefined as two separate species, Torque teno sus virus 1 (TTSuV1) and 2 (TTSuV2), due to the low sequence identity shown [9,10]. The presence of TTSuV1 has been investigated in a number of countries in Europe, North America and Asia with prevalences ranging between 9-100% in the studied populations [6,7,11-14]. Studies of TTSuV2 have shown prevalences in the same range [7,13-15]. TTSuV has been demonstrated in European wild boar [16], and co-infection with both TTSuV1 and 2 is a common finding, in wild boar as well as in domestic pigs.

Thus, studies clearly show that TTSuV1 and TTSuV2 are widely distributed in domestic pigs in America, Europe and Asia, but the situation in domestic pig populations on the African continent is not known. In this pilot study we performed a first investigation of TTSuV1 and 2 distributions in Uganda.

## Results

### Detection of TTSuV1 and TTSuV2

**Figure 1:**
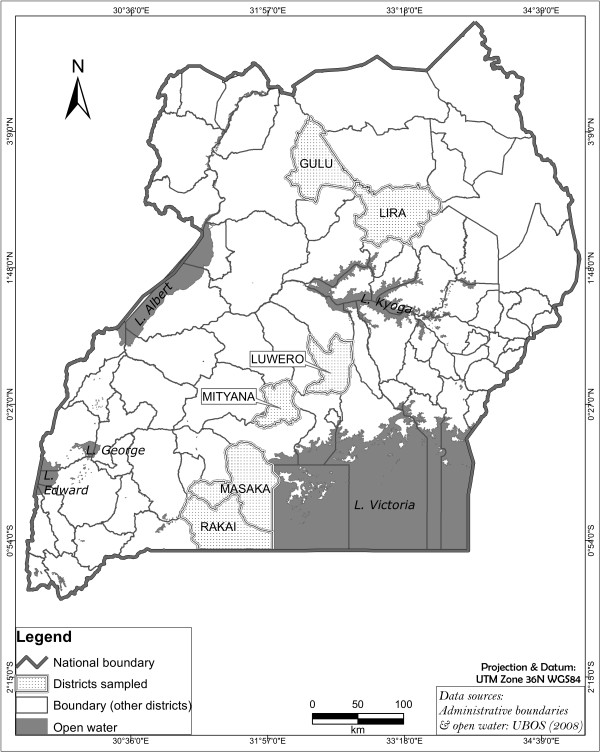
**Map of Uganda**. Map of Uganda showing the six districts where the samples were collected.

TTSuV was detected in pigs from all the six different districts (Figure 1) investigated and in total TTSuV was detected in 51.6% (49/95 samples positive for either of viruses or both) of the serum samples. TTSuV1 was found in 16 out of 95 samples (16.8%) and TTSuV2 in 46 out of 95 samples (48.4%). Co-infection with both species was seen in 13 out of 95 samples (13.7%).

### Genetic and phylogenetic studies of TTSuV1 and 2 UTR sequences

The sequences from the PCR products of 14 TTSuV1 and 32 TTSuV2 positive serum samples were used for diversity and phylogenetic studies. All the sequences have been deposited in the GenBank: accession number JN191451-96.

**Figure 2:**
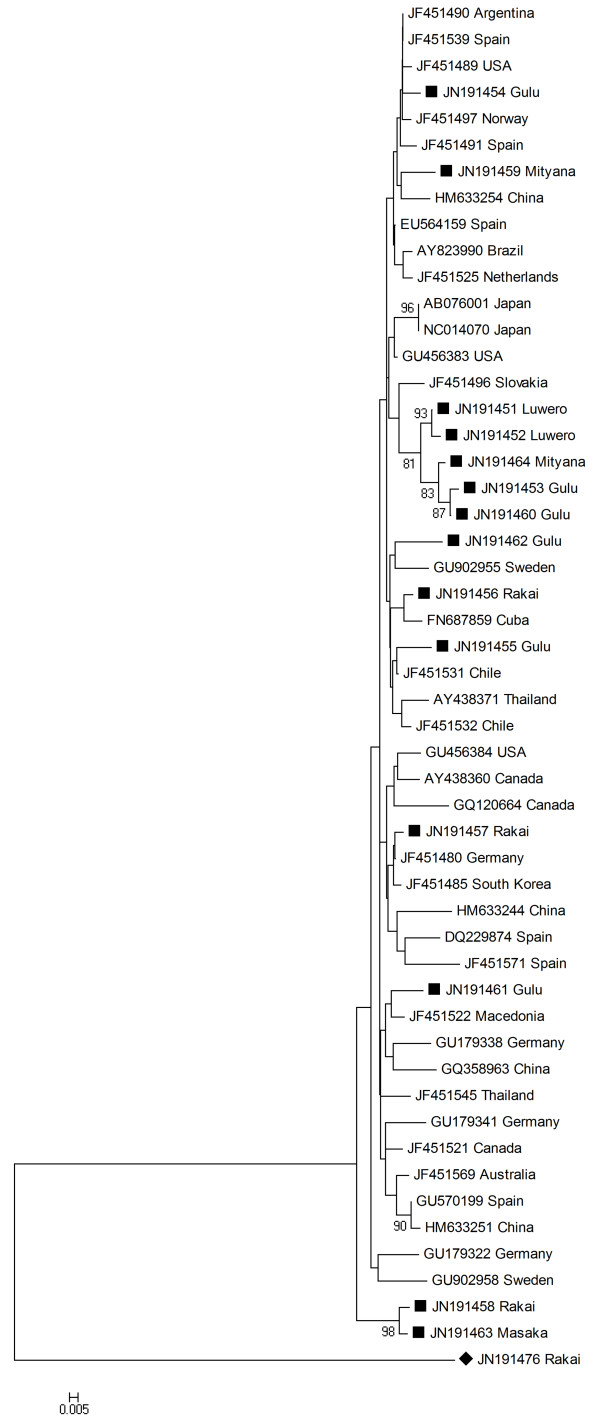
**Phylogenetic analyses - TTSuV1**. Phylogenetic analysis of TTSuV1 based on a 212 nt long sequence from the 5' UTR region, using Neighbour-joining algorithm and 1,000 bootstraps. Only bootstrap values above 70% are shown. The TTSuV1 sequences from this study are marked with ■ and the one marked ♦ is a TTSuV2 sequence from this study used as an outgroup. The district from where the samples from this study originated is indicated after the accession number.

The TTSuV1 sequences in this study showed a sequence similarity of 90-100% to each other. When compared to 38 TTSuV1 sequences originating from other parts of the world, the identity ranged from 90 to 98%. The phylogenetic analysis of the TTSuV1 sequences (Figure 2) shows no unique geographical clustering of the Ugandan sequences.

**Figure 3:**
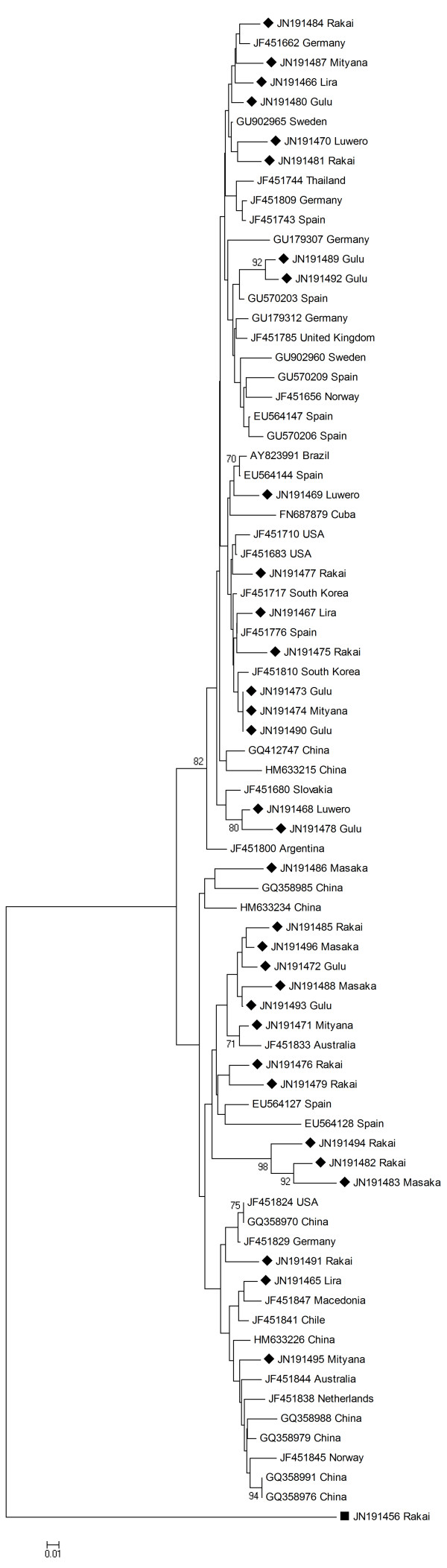
**Phylogenetic analyses - TTSuV2 Phylogenetic analysis of TTSuV2 based on a 185 nt long sequence from the 5' UTR region, using Neighbour-joining algorithm and 1,000 bootstraps**. Only bootstrap values above 70 are shown. The TTSuV2 sequences from this study are marked with **♦ **and the one marked **■ **is a TTSuV1 sequence from this study used as an outgroup. The district from where the samples from this study originated is indicated after the accession number.

The Ugandan TTSuV2 sequences displayed a higher sequence divergence with pair wise similarities between 63-100%. When compared to 44 TTSuV2 sequences from America, Asia and Europe the pair wise similarities ranged between 64-99%. No clear geographical grouping was seen (Figure 3). The phylogenetic tree displayed two main clades, with separation due to an 11-12 nt long region present in the sequences forming one of the clades. Within this group three of the Ugandan sequences (JN191494, JN191482 and JN191483) had an additional 20 nt long region a bit further downstream from the first region and these do therefore group on a well-supported sub-clade.

## Discussion

This is the first study investigating the presence of TTSuV1 and 2 in domestic pigs in Uganda, and indeed on the African continent. Available studies from many parts of the world demonstrate that these viruses are widespread among pig populations [3,17], and it is therefore not surprising to see the same pattern in Ugandan pigs: our study showed that around half of the investigated domestic pigs were infected with either of the TTSuV species, indicating that TTSuV infection is common in Uganda. TTSuV1 was detected in fewer pigs compared to TTSuV2, and in most (13/16) of the samples positive for TTSuV1, TTSuV2 was also detected. Whereas TTSuV2 was detected in similar frequencies as in previous comparable studies [12,18,19], the detected prevalence of TTSuV1 positive pigs was lower compared to what has been reported elsewhere [11,12,19,20].

The genetic similarity of the sequenced Ugandan TTSuV UTR regions was higher for TTSuV1 than for TTSuV2 (TTSuV1: 90-100%; TTSuV2: 63-100%). The sequence identity to sequences from other parts of the world displayed a similar pattern (TTSuV1: 90-98%; TTSuV2: 64-99%). Previous studies have shown that TTSuV lacks an apparent geographical clustering when studying the UTR region [11]. The phylogenetic analysis of TTSuV1 (Figure 2) displays the Ugandan sequences evenly distributed among the isolates from other parts of the world. TTSuV2 also grouped evenly together with the sequences from other parts of the world (Figure 3). A grouping of the sequences into two clades was due to an 11-12-nucleotide long region present in a number of sequences (a similar result was obtained by using Maximum Likelihood based on the Tamura-Nei model (data not shown)). An additional 20-nucleotide long region was present in three of the Ugandan isolates and these therefore formed a well-supported sub-clade. Overall, the bootstrap values within the clades were generally low for both genotypes, meaning that branching patterns of these are not always highly confident. Using the ORF1 capsid gene as a marker, which has been suggested previously [7], could possibly give a better idea of the evolutionary origin of the sequences, since the capsid gene presumably is under purifying selection. Also using the complete genome would be valuable for phylogenetic analysis [9,10] to investigate possible episodes of viral introduction(s) and relations to epidemiological events.

The potential pathogenicity of TTSuV is unclear, but it is speculated that these viruses can be directly or indirectly involved in disease development. Several studies in pigs concern the role of co-infection of TTSuV1 and 2 with known pathogenic viruses like PCV-2, the causative agent of PMWS [13,19,21]. Although two of these studies indicate a somewhat higher co-infection rate of TTSuV in diseased pigs further studies are needed to prove the involvement of TTSuV in PMWS pathogenesis. 18 out of the 95 samples in the present study had previously been tested positive for African swine fever virus, but the proportion of TTSuV1 and 2 positives among these 18 samples did not differ significantly from that of the entire sample (data not shown).

## Conclusion

In conclusion, the present study is the first in assessing the presence of TTSuV in domestic pigs on the African continent. In this limited study we have shown that, like in other parts of the world, TTSuV1 and 2 seem to be widespread among domestic pigs in Uganda.

## Methods

### Samples

Serum samples from 95 domestic pigs in Uganda were used in this study. The samples were collected from 70 pig farms in six different districts in Uganda - Gulu, Lira, Luwero, Masaka, Mityana and Rakai (Figure 1) during the years 2010 and 2011, as part of an in-depth research project on African swine fever epidemiology. Half of the samples originated from farms with suspected outbreaks of African swine fever, and half from farms with no clinical signs of the disease. The Ministry of Agriculture Animal Industry and Fisheries together with Makerere University are mandated to carry out animal disease investigations in the country. This is done by veterinarians who handle the animals under internationally recognized guidelines.

### DNA extraction

The majority of the serum samples were extracted using DNeasy Blood & Tissue kit (Qiagen, Hilden, Germany) according to the protocol "Purification of Total DNA from Animal Blood or Cells" provided by the manufacturer. 200 microliter serum was used from each sample and the DNA was eluted in 100 μl AE buffer. A few of the samples were extracted through combining TRIzol (Invitrogen, Carlsbad, CA, USA), back extraction buffer (4 M Guanidine Thiocyanate, 50 mM Sodium Citrate, 1 M Tris) and QIAamp DNA mini kit (Qiagen, Hilden, Germany). After removing the RNA phase from the serum/TRIzol/chloroform mixture, 750 μl back extraction buffer was added. The samples were then mixed by inversion for 3 min and centrifuged for 30 min at 12 000 × g. The aqueous upper phase containing DNA was transferred to a new tube and mixed with the same volume 70% ethanol before loading it onto a DNeasy Mini Spin Column and centrifuged at 12 000 × g for 30 s. The column was washed with 500 μl AW1 and AW2 buffer, respectively, and the DNA was finally eluted in 50 μl AE buffer. The extracted DNA was stored in -20 C until further use.

### TTSuV1 and 2 detection and partial sequencing

TTSuV1 and TTSuV2 were detected by PCR using primers specific for the UTR of the respective viral genome [7]. Each reaction consisted of 1× PCR buffer, 2.5 mM MgCl_2_, 1.0 mM dNTP, 0.4 μM forward primer and reverse primer each, and 1.25 U AmpliTaq Gold DNA polymerase (Applied Biosystems, Foster City, CA, USA). Two or four μl DNA, depending on the DNA concentration achieved from the two different extraction methods, was used as template. Amplification was performed with the following reaction conditions: a 12 min enzyme activation step at 95 C followed by 39 cycles of 95 C for 30 s, 58 C for 30 s and 72 C for 90 s, finishing with one cycle for 10 min of 72 C. The PCR products were visualized on a 1.3% agarose gel. The PCR positive products were purified using the QIAquick PCR purification kit (Qiagen, Hilden, Germany) according to the manufactures' instructions and eluted in 30 μl EB. The purified products were sent to Macrogen Inc. (Seoul, Korea) for Sanger sequencing.

### Phylogenetic studies

The chromatograms were edited in SeqMan (Lasergene 9, DNASTAR Inc., Madison, USA). The edited sequences were subsequently aligned by ClustalW in BioEdit http://www.mbio.ncsu.edu/bioedit/bioedit.html and the sequence identity was compared. The phylogenetic relationship among the TTSuV1 and TTSuV2 sequences from this study were compared to previously published sequences available from GenBank http://www.ncbi.nlm.nih.gov/genbank using Mega 5 [22] for the construction of a phylogenetic tree using the Neighbour-joining algorithm with the p-distance substitution model and with a bootstrap value of 1000.

## Competing interests

The authors declare that they have no competing interests.

## Authors' contributions

MaB has performed the laboratory experiments and contributed to the data analysis and drafting of the manuscript. KS and CM coordinated the field work and contributed to study design, data analysis and final manuscript preparation. ARO were involved in field work and final manuscript preparation. MiB contributed to study design, data analysis and final manuscript preparation. ALB contributed to laboratory analysis, study design, data analysis, manuscript draft and final manuscript preparation. All authors have read and approved the final manuscript.
